# Unveiling the Influence of the Antioxidant System in Eucalyptus Seedlings in the Face of Adequate Water Availability

**DOI:** 10.3390/plants14213405

**Published:** 2025-11-06

**Authors:** Ricardo Gava, Dthenifer Cordeiro Santana, Cid Naudi Silva Campos, Ana Carina da Silva Cândido Seron, Larissa Pereira Ribeiro Teodoro, Mayara Fávero Cotrim, Regimar Garcia dos Santos, Renato de Mello Prado, Rafael Felippe Ratke, Marcia Leticia Monteiro Gomes, Paulo Eduardo Teodoro

**Affiliations:** 1 Câmpus of Chapadão do Sul, Universidade Federal de Mato Grosso do Sul, UFMS, Chapadão do Sul 79560-000, Brazil; ricardo.gava@ufms.br (R.G.); dthenifer.santana@ufms.br (D.C.S.); ana.candido@ufms.br (A.C.d.S.C.S.); larissa_ribeiro@ufms.br (L.P.R.T.); mayara.cotrim@unesp.br (M.F.C.); rafael.ratke@ufms.br (R.F.R.); marcia_leticia@ufms.br (M.L.M.G.); paulo.teodoro@ufms.br (P.E.T.); 2 Department of Horticulture, The University of Georgia, Plant Sciences Building, Athens, GA 30602, USA; regimar.garcia@uga.edu; 3 Department of Soil Science, São Paulo State University “Júlio de Mesquita Filho” UNESP/FCAV, Jaboticabal 14884-900, Brazil; rm.prado@unesp.br

**Keywords:** photosynthesis, abiotic stresses, drought tolerance

## Abstract

The study of the relationship between water availability, photosynthetic behavior, flavonoid accumulation, and antioxidant response offers new perspectives for enhancing nursery practices, resulting in more vigorous eucalyptus seedlings that are tolerant and have greater potential for field establishment. Under the hypothesis that different eucalyptus genetic materials show contrasting responses to water availability in the soil–plant–atmosphere system, this study aims to evaluate the physiological behavior of clones subjected to different irrigation intervals, with an emphasis on the role of flavonoids as antioxidants in mitigating the effects of water stress. The experimental design was structured in strips containing five eucalyptus clones and irrigation with different watering intervals: 1, 2, 4, and 8 days. Evaluations of net photosynthesis, transpiration, and instantaneous water use efficiency were performed. In addition to the physiological assessments, the flavonoids daidzein, genistein, and genistin were determined. Clones C1, C2, and C3 excelled in photosynthesis and transpiration at 2- and 4-day intervals, while C1 and C2 maintained superior performance even at an 8-day interval. WUE was highest in C5 and increased with water stress, showing a quadratic fit in all clones. Regarding flavonoid production, C1 and C3 showed greater daidzein accumulation, with a quadratic response to the withdrawal interval. Genistein showed a linear reduction only in C2, while genistein increased in C1, peaking around 11 days. Eucalyptus clones exhibit distinct physiological and biochemical responses to variations in irrigation intervals. More frequent irrigation favors photosynthetic activity and transpiration, particularly in clones C1, C2, and C3, whereas longer irrigation intervals reduce these processes but enhance water use efficiency, especially in C5.

## 1. Introduction

In recent years, Brazil has emerged as a leading center for forest product development, particularly from species of the genus Eucalyptus (Myrtaceae) introduced into the country for its high potential for timber and pulp production [1], eucalyptus contributes substantially to the forestry economy, accounting for approximately 12% of global charcoal production [2]. Owing to its rapid growth and high adaptability, it is widely used in reforestation programs, although its cultivation is constrained by various biotic and abiotic factors [3]. Owing to its rapid growth and high adaptability, it is widely used in reforestation programs, although its cultivation is constrained by various biotic and abiotic factors. Given its economic and environmental importance, understanding the factors that limit production and early performance of eucalyptus seedlings is essential.

Among the most critical challenges for seedling production and subsequent plant development is water scarcity. This abiotic stress compromises initial seedling establishment, directly affecting growth and acclimation capacity. Soil water deficit elicits chemical signaling from roots to leaves via the xylem, promoting stomatal closure and reducing CO_2_ assimilation [4], which in turn lowers photosynthetic rates, suppresses growth, and increases plant vulnerability [5]. Although studies on eucalyptus responses to water stress exist, most focus on advanced growth stages or field conditions, leaving limited information on the physiological and morphological impacts of water deficit on seedlings, particularly across varying stress intensities and durations. This gap limits understanding of early adaptive mechanisms and underscores the need for studies characterizing the initial responses of eucalyptus seedlings to water limitation.

Plant tolerance to environmental stressors depends on intrinsic physiological and biochemical capacities. Among tolerance mechanisms to environmental stress, the production of secondary metabolites is prominent, as these compounds participate in essential regulatory processes under stress [6]. These metabolic responses help prevent cellular damage by stabilizing chlorophyll and membranes, thereby limiting solute leakage and loss of structural integrity [7]. Among these compounds, flavonoids play a fundamental role as antioxidant defense, preventing the destruction of the photosynthetic apparatus, damage to the cell membrane, protein denaturation, and plant growth inhibitors [8]. In eucalyptus, water stress significantly alters flavonoid production and profiles, reflecting metabolic adjustments that mitigate oxidative damage [3]. However, most research has focused on mature plants or narrow case studies, with limited investigation of seedlings and of integrated responses across flavonoid classes under controlled water-deficit conditions. Thus, elucidating how water stress modulates flavonoid biosynthesis in eucalyptus seedlings is essential to clarify tolerance mechanisms at early stages and to inform management strategies and indirect genetic improvement for water resilience.

In this context, strategies that integrate appropriate water management with metabolic enhancement are essential for producing more resilient eucalyptus seedlings. Bioactive compounds such as flavonoids are central to this process, functioning not only as antioxidants but also as modulators of physiological signaling related to defense and development.

Therefore, the primary objective of this study was to investigate relationships among water availability, photosynthetic behavior, flavonoid accumulation, and antioxidant responses in eucalyptus seedlings, with the aim of understanding how these mechanisms interact to mitigate water stress. As a secondary objective, the physiological behavior of different genotypes under varying irrigation intervals was evaluated to identify materials with greater adaptive capacity and biochemical potential under water limitation.

## 2. Results

According to the analysis of variance (Table 1), a significant interaction between the clone and irrigation interval factors was observed for all variables analyzed, except for water use efficiency (WUE), which showed a significant effect only independently for each factor. This indicates that the variation in WUE is explained separately by genetic material and irrigation regime, with no interaction between them.

**Figure 1:**
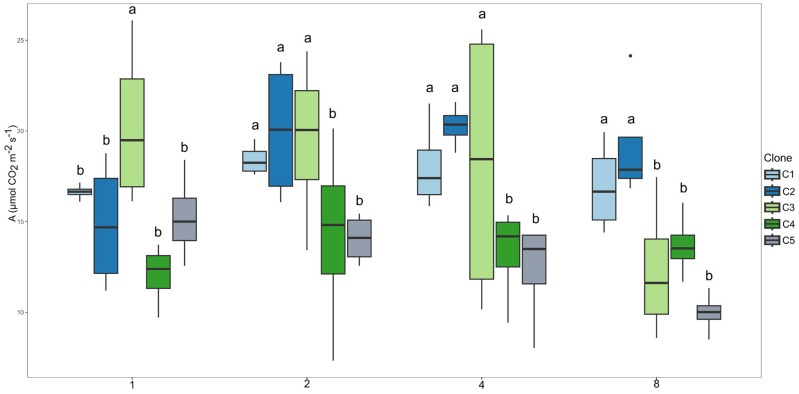
Clustering of means by the Scott–Knott test for clones within each irrigation interval for net photosynthesis (µmol CO_2_ m^−2^ s^−1^). Means followed by the same letter do not differ at 5% probability level.

In the first irrigation interval, clone C3 exhibited the highest photosynthetic rate (Figure 1). In the second regime, clones C1, C2, and C3 presented the highest mean values. A similar performance pattern was observed under the 4-day irrigation interval. Under the 8-day regime, only clones C1 and C2 stood out.

**Figure 2:**
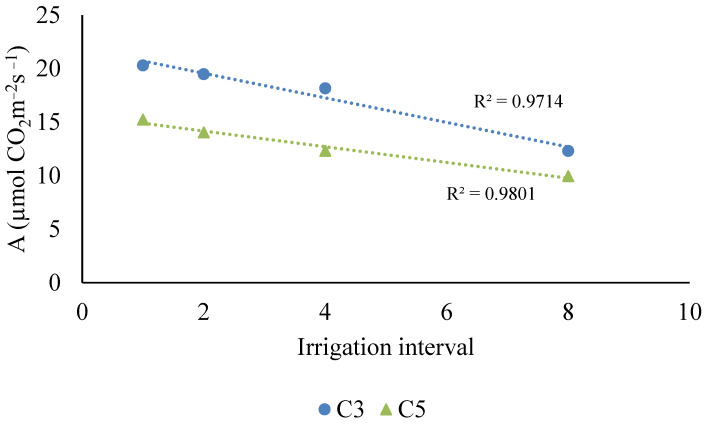
Regression models of net photosynthesis (µmol CO_2_ m^−2^ s^−1^) as a function of irrigation interval for clones C2, C3, and C5.

For the quantitative factor (irrigation cycle), regression analysis was performed for the clones that showed a significant effect: C3 and C5. Both clones exhibited a progressive reduction in photosynthetic rate as the irrigation interval increased (Figure 2).

**Figure 3:**
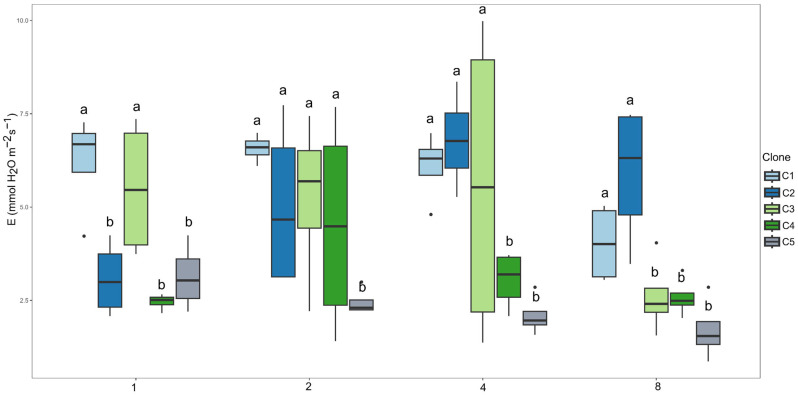
Clustering of means by the Scott–Knott test for clones within each irrigation interval for transpiration (E, mmol H_2_O m^−2^ s^−1^). Means followed by the same letter do not differ at 5% probability level.

Regarding transpiration (E, Figure 3), clones C1 and C3 showed the highest rates in the first irrigation interval. In the second interval, clones C1, C2, C3, and C4 exhibited the highest mean values. In the 4-day irrigation interval, clones C1, C2, and C3 maintained the highest values, whereas in the 8-day interval, clones C1 and C2 stood out.

Regarding the quantitative factor, clones C2 and C3 showed distinct behavior depending on the irrigation interval, both adjusting to the quadratic model. Clone C2 reached its maximum transpiration with irrigation close to the tenth day, while clone C3 peaked with approximately 5 days between irrigation. Both clones exhibited a decrease in transpiration as the irrigation interval increased.

**Figure 4:**
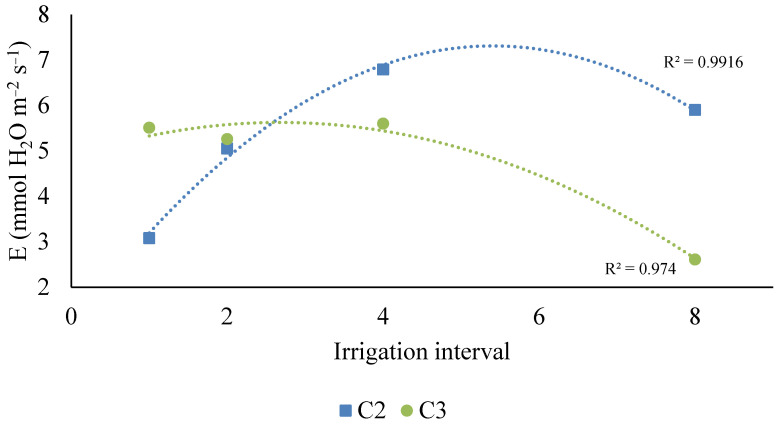
Regression models for transpiration (E, mmol H_2_O m^−2^ s^−1^) as a function of the irrigation interval for clones C2 and C3.

**Figure 5:**
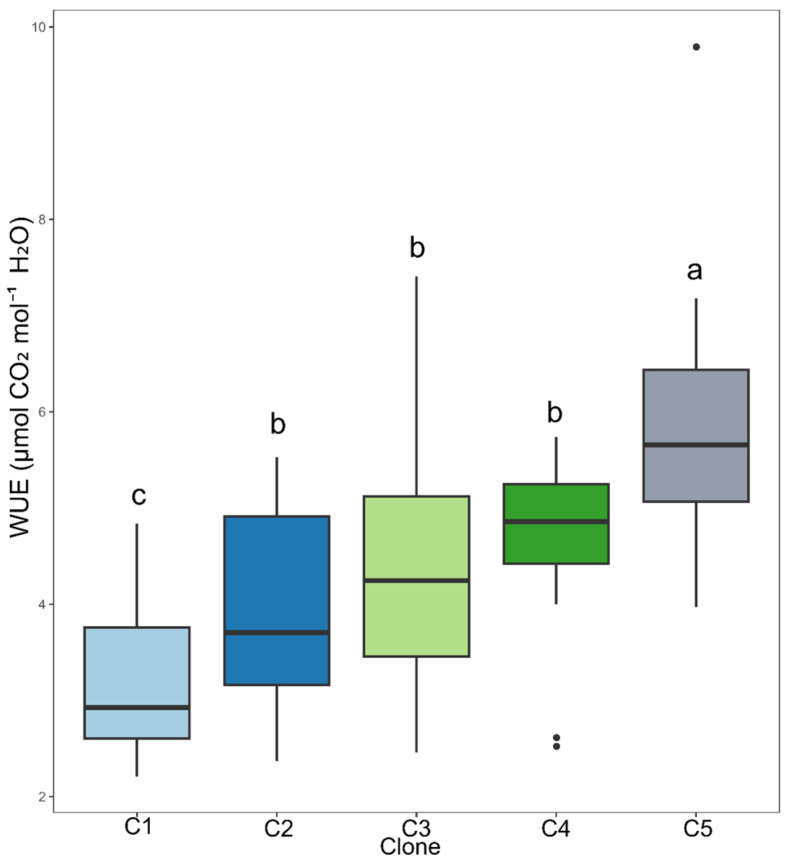
Clustering of means by the Scott–Knott test for clones for instantaneous water use efficiency (WUE, μmol CO_2_ mol^−1^ H_2_O). Means followed by the same letter do not differ significantly.

The genetic material that showed the highest instantaneous water use efficiency (WUE, Figure 4) was clone C5, followed by clones C2, C3, and C4, which exhibited similar mean values, indicating intermediate performance (Figure 5). These results suggest that clone C5 has a greater capacity to maintain the balance between carbon assimilation and water loss through transpiration, even under varying irrigation intervals.

Considering the quantitative factor (irrigation interval), a mean quadratic fit was observed for WUE, with an upward trend as the interval between irrigations increased. This trend represents the general behavior of the variable as a function of water availability, irrespective of genetic material. This response may reflect an adaptive mechanism to moderate water restriction, characterized by partial stomatal closure that reduces transpiration without markedly compromising carbon assimilation.

**Figure 6:**
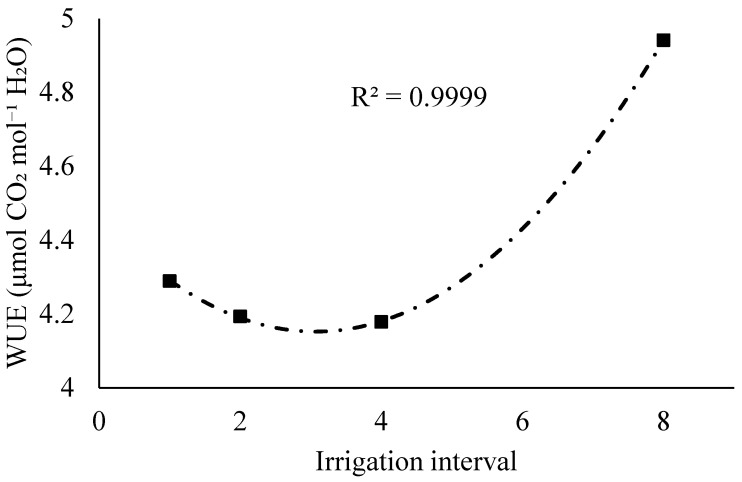
Regression models for instantaneous water use efficiency (WUE, μmol CO_2_ mol^−1^ H_2_O) as a function of the irrigation interval for the clones.

Regarding daidzein content (Figure 6), clone C3 had the highest mean in the first irrigation interval. During the 2-day irrigation interval, clones C1 and C3 exhibited the highest values. For the 4-day interval, clone C1 maintained the highest mean, a pattern that was also repeated in the 8-day interval.

**Figure 7:**
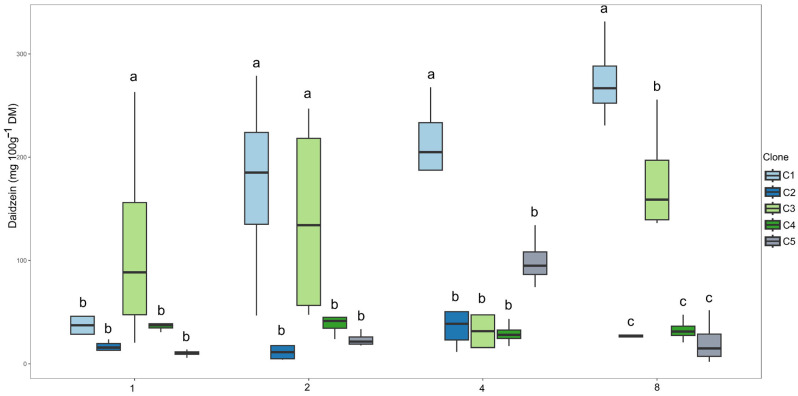
Clustering of means by the Scott–Knott test for clones within each irrigation interval for daidzein content (mg 100 g^−1^ DM). Means followed by the same letter do not differ significantly.

**Figure 8:**
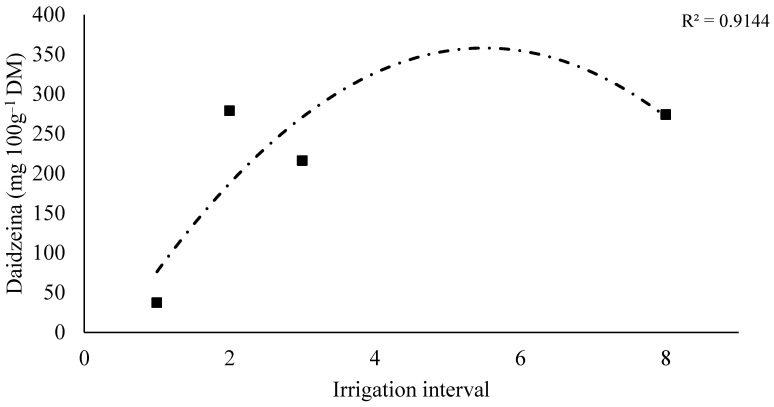
Regression models for aidzein (mg 100 g^−1^ DM) as a function of irrigation interval for clones C1 and C3.

Considering the quantitative factor (irrigation interval), analysis of daidzein as a response variable indicated a significant effect only for clone C1 (Figure 7). Clone C1 exhibited increasing daidzein levels with longer intervals between irrigations, with the derivative indicating a local maximum near the 4-day irrigation interval (Figure 8).

**Figure 9:**
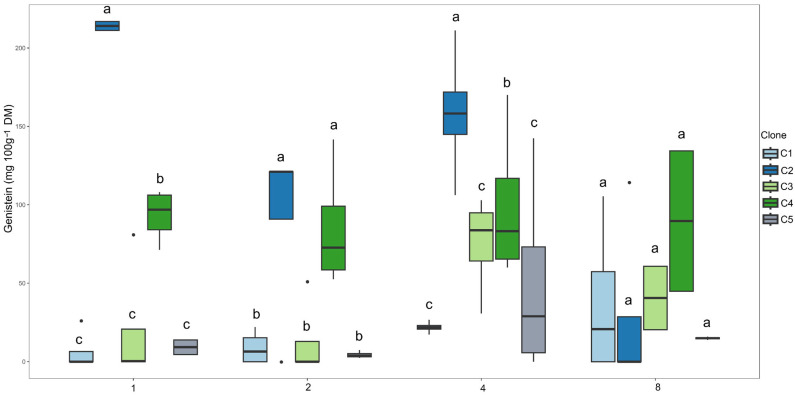
Clustering of means by the Scott–Knott test for clones within each irrigation interval for genistein content (mg 100 g^−1^ DM). Means followed by the same letter do not differ significantly.

Clone C1 had the highest genistein content in the 1-, 2-, and 4-day irrigation intervals (Figure 9). During the 8-day irrigation interval, all clones exhibited similar mean values, suggesting a possible stabilizing effect of increased water restriction on the production of this flavonoid.

**Figure 10:**
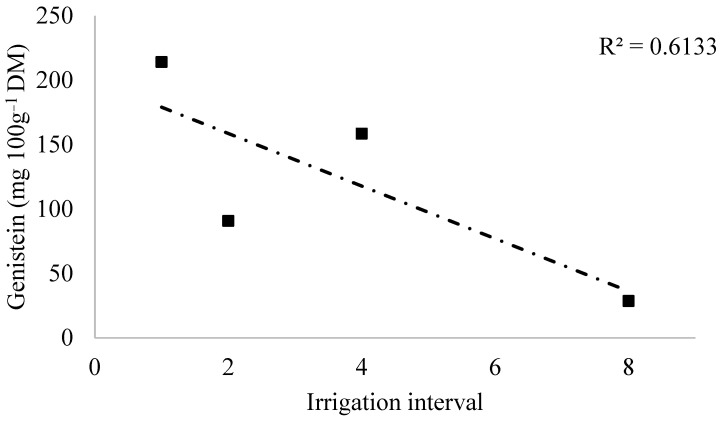
Regression models for genistein content (mg 100 g^−1^ DM) as a function of the irrigation interval for clone C2.

Regarding the quantitative factor (irrigation interval), only clone C2 showed significant variation in genistein content, which was best described by a decreasing linear model (Figure 10). This indicates that, for this clone, the increase in the interval between irrigations resulted in a progressive reduction in genistein synthesis, possibly due to the limitation of metabolic activity under prolonged water stress.

**Figure 11:**
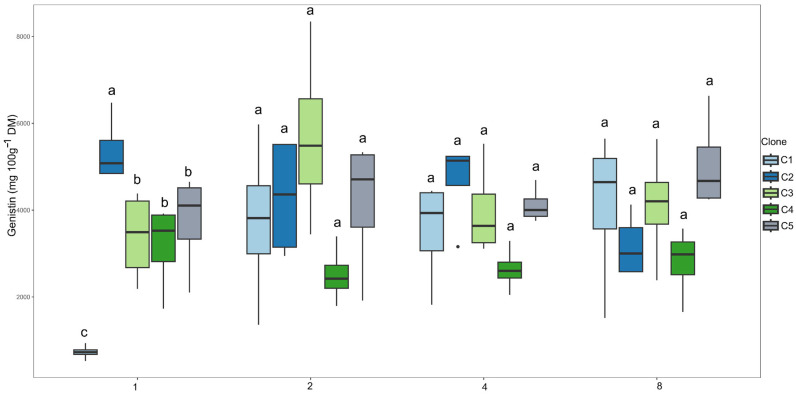
Clustering of means by the Scott–Knott test for clones within each irrigation interval for genistin content (mg 100 g^−1^ DM). Means followed by the same letter do not differ significantly.

For the compound genistin, clone C2 showed the highest mean in the 1-day irrigation interval (Figure 11). In the other irrigation intervals, all clones had similar means, suggesting that under conditions of greater water availability, clone C2 is more responsive in the production of this flavonoid. There was no fitting for the regression curves.

**Figure 12:**
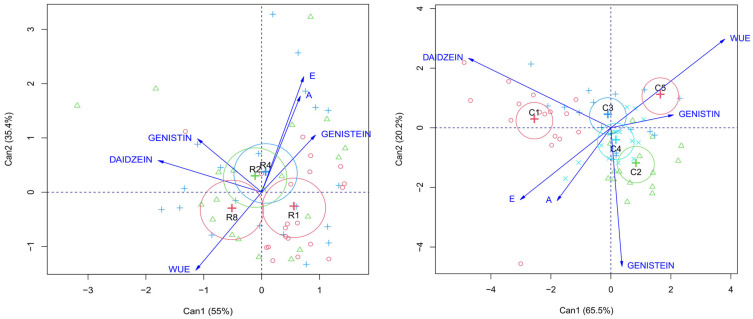
Canonical variables showing correlations between physiological parameters (A: net photosynthesis, E: transpiration, WUE: water use efficiency) and phenolic compounds (daidzein, genistein, and genistin) under each evaluated irrigation interval: R1 = 1 day; R2 = 2 days; R4 = 4 days; and R8 = 8 days.

A multivariate analysis of canonical variables was performed to investigate the interrelationship between physiological parameters and biochemical compounds as a function of irrigation intervals and different clones (Figure 12). This approach allowed the integration of the effects of the evaluated factors and the visualization of groupings and correlations among variables. Regarding the irrigation interval, it was observed that A and E, as well as genistein, showed a strong association with irrigation intervals R2 and R4, which is in agreement with previous results indicating that these intervals are favorable for photosynthetic activity and for the production of this flavonoid. On the other hand, water use efficiency (WUE) showed the opposite association, with a stronger relationship with the 8-day irrigation interval, reinforcing the findings that indicate increased efficiency under greater water restriction. Still in relation to the irrigation interval factor, genistin and daidzein showed a higher correlation with R2, suggesting that the production of these compounds is favored by more frequent, but not excessive, irrigation.

Regarding the genetic factor, genistin and WUE showed a stronger association with clone C5, reflecting its prominence in water efficiency and flavonoid production under certain conditions. Daidzein, in turn, correlated mainly with clones C1 and C3, which had already shown greater accumulation of this compound in intermediate and long intervals. Net photosynthesis (A) and transpiration (E) were more strongly associated with clone C4, indicating that this material maintained greater physiological activity, although not necessarily the highest accumulation of secondary metabolites.

**Figure 13:**
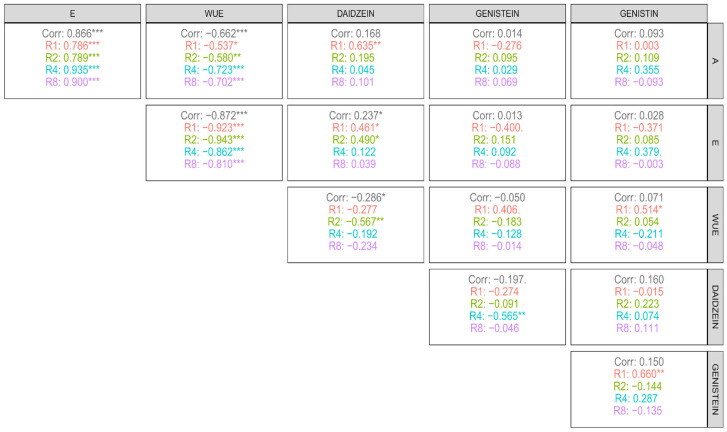
Correlations between physiological variables (A: net photosynthesis, E: transpiration, WUE: water use efficiency) and phenolic compounds (daidzein, genistein, and genistin) under each evaluated irrigation interval: R1 = 1 day; R2 = 2 days; R4 = 4 days; and R8 = 8 days. *, **, ***: significant at 5%, 1%, and 0.1% respectively by the t-test.

As for the correlations between physiological and biochemical variables as a function of irrigation intervals (Figure 13), a high positive correlation was observed between transpiration (E) and photosynthetic rate (A) across all intervals, with coefficients above 0.78, indicating that increased CO_2_ assimilation is directly associated with intensified water loss through the leaves, reflecting a strong dependence between physiological functions. Water use efficiency (WUE) exhibited a high negative correlation with A and E, which reinforces previous results indicating that WUE tends to increase when there is greater water restriction, under conditions where A and E decrease. In addition, WUE showed a moderate negative correlation with daidzein (r = −0.567), suggesting that high levels of this flavonoid occur under conditions of lower water efficiency. On the other hand, WUE showed a moderate positive correlation with genistein (r = 0.406) and genistin (r = 0.514), indicating that these compounds may be associated with more efficient water use strategies, possibly through cellular protection mechanisms under stress.

Daidzein, in turn, showed a high correlation with A (r = 0.635) in the first irrigation interval and a moderate positive correlation with E in the first two intervals (values around 0.4), indicating that its production is related to conditions of greater physiological activity. Also, in this same interval (first interval), daidzein correlated positively with genistein and genistin, suggesting that, under high water availability, there is a joint stimulus in the production of these isoflavones.

**Figure 14:**
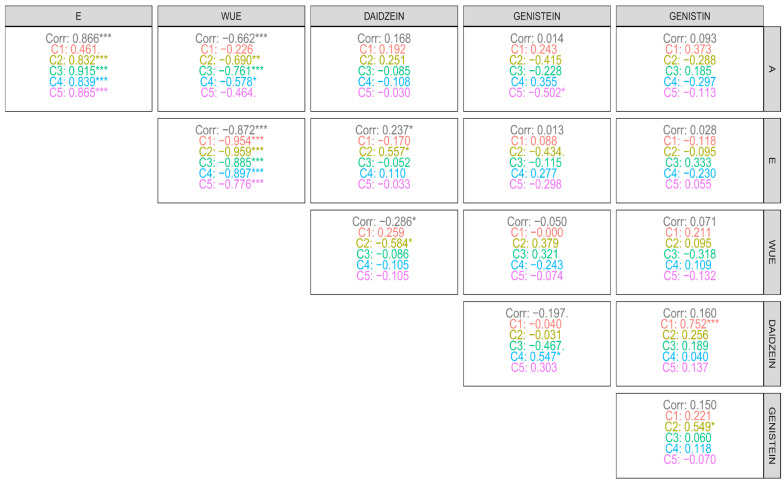
Correlations between physiological variables (A: net photosynthesis, E: transpiration, WUE: water use efficiency) and phenolic compounds (daidzein, genistein, and genistin) under each evaluated irrigation interval: C1, C2, C3, C4, and C5 indicate the clones evaluated. *, **, ***: significant at 5%, 1%, and 0.1% respectively by the t-test.

Regarding the correlations between variables within each clone (Figure 14), a pattern of high positive correlation was observed between photosynthetic rate (A) and transpiration (E) in almost all genetic materials (r > 0.80), except for clone C1, which showed a lower correlation. The correlation between A and WUE was strongly negative in most clones, except in C1, where this relationship was weak, and in C5, where the correlation was moderately negative, suggesting a possible greater physiological stability in these genotypes. Isoflavones, in general, did not show significant correlations with A, indicating that the production of these secondary metabolites is not directly associated with the photosynthetic rate under the evaluated conditions. The variable E, in turn, showed strongly negative correlations with WUE in all clones, indicating an opposite effect between water loss and efficiency in its use. Among the isoflavones, daidzein exhibited a positive, high-magnitude correlation with E only in clone C2, suggesting that transpiration may contribute to the greater synthesis of this compound in this material. In the other clones, this relationship was not significant.

Furthermore, no relevant correlations were observed between E and the other isoflavones (genistein and genistin). When analyzing the correlations among the phenolic compounds themselves, the only ones of greater relevance were those between genistein and daidzein in C4 (positive correlation), between daidzein and genistin in C1, and between genistin and genistein in C2, all of which were also positive. These results highlight that, in certain genetic materials, there is a combined regulation of the production of these flavonoids, possibly linked to specific environmental response mechanisms in the genetic material.

Eucalyptus clones showed different responses to the irrigation intervals. Clones C1, C2, and C3 stood out in photosynthesis and transpiration in the 2- and 4-day intervals, while C1 and C2 maintained superior performance even with an 8-day interval. Clones C2 and C3 exhibited quadratic behavior for photosynthesis and transpiration, with peaks occurring at intermediate intervals, whereas clone C5 showed a linear decline in photosynthesis. WUE was highest in C5 and increased with water stress, showing a quadratic adjustment in all clones.

In flavonoid production, C1 and C3 showed greater accumulation of daidzein, with a quadratic response to their irrigation interval. Genistein showed a linear reduction only in C2, while genistin showed an increase in C1, with a peak occurring close to 11 days. Multivariate analysis indicated that A, E, and genistein were associated with 2- and 4-day irrigation intervals, while WUE correlated with the 8-day interval. The correlations showed a strong association between A and E in all clones, with WUE negatively correlated with these variables. Isoflavones exhibited specific correlations among themselves in certain clones, particularly between genistein, genistin, and daidzein.

## 3. Discussion

Plants within the same species behave differently when subjected to stress, whether biotic or abiotic. This distinction is due to the different evolutionary processes that genetic materials have undergone over time, such as various strategies for coping with drought, which is a significant selection factor in breeding programs [9] and in recommendations for such clones in specific environments. In general, plant productivity and drought tolerance are inversely proportional variables, and the forms of adaptation result in different responses by genetic materials in the face of water limitation [10].

Eucalyptus clones exhibited distinct physiological and biochemical responses to different irrigation intervals, reflecting variations in both carbon assimilation and phenolic compound synthesis [11]. Overall, more frequent irrigation (1–4 days) favored higher photosynthetic and transpiration rates, especially in clones C1, C2, and C3. Clone C2 exhibited higher photosynthetic and transpiration rates under the longer irrigation interval (around 8 days), a pattern that initially appears to contradict the expectation of reduced transpiration under water limitation. This response is consistent with moderate stress, under which plants maintain partial stomatal conductance that balances carbon assimilation with water loss. Such behavior indicates an adaptive tolerance mechanism involving dynamic adjustments in stomatal aperture and photosynthetic efficiency, sustaining CO_2_ assimilation without a precipitous decline in transpiration.

The response of clone C2 reflects resilience and physiological plasticity under partial reductions in water availability. More widely spaced irrigation intervals reduced biochemical process rates but increased water use efficiency (WUE), especially in clone C5. This behavior is consistent with the expected physiological response to moderate water stress, in which partial stomatal closure conserves water use.

Stomatal regulation prioritizes carbon assimilation per unit water over maximizing absolute transpiration [12]. This functional maintenance can be provided by several characteristics of the genetic material, such as deeper or more adaptable root systems, greater stomatal sensitivity to water stress, and favorable proportions of water and nutrient transport that allow sustained growth under conditions of moderate drought [11,13]. These results suggest that the C5 clone may be particularly suitable for environments with limited water availability, as it exhibits good physiological performance while also achieving water savings.

The expression of secondary metabolites in response to water availability is a key factor in enabling plants to adapt and evolve under various stress conditions [14]. Regarding flavonoid production, daidzein exhibited quadratic behavior in clones C1 and C3, with greater accumulation at intermediate intervals, indicating that water stress can stimulate the synthesis of secondary compounds as a plant defense mechanism. Genistein exhibited a linear reduction in C2 as the irrigation interval increased, indicating sensitivity to more prolonged water limitation, like the behavior observed in photosynthesis and transpiration. Genistin showed a significant increase in clone C1, with an estimated peak at around 11 days, indicating that the accumulation of this compound may also be favored by moderate stress intensity. Secondary metabolites play a crucial role in the detoxification of reactive oxygen species (ROS) during stress. As photosynthesis is highly sensitive to ROS accumulation in plants, most photosynthetic enzymes are preferential targets for oxidation [15].

Genistein is a phytochemical classified as an isoflavone, whose biosynthesis in plants begins with the shikimic acid pathway, leading to the phenylpropanoid pathway and, subsequently, the flavonoid pathway [16]. Phenylalanine Ammonia Lyase (PAL) is the first enzyme and a crucial regulatory point in the phenylpropanoid pathway. It is responsible for the conversion of L-phenylalanine to cinnamic acid. Water stress, caused by long irrigation intervals, is an abiotic stressor known to affect secondary metabolism many stresses increase PAL activity as a defense mechanism, severe or prolonged water stress can result in a generalized decrease in metabolic activity and several enzymes due to low water availability, thus reducing the flow of precursors into the pathway [17]. Reducing PAL activity would limit the production of initial precursors. Chalcone Synthase (CHS) catalyzes the first committed step in the flavonoid biosynthesis pathway, the condensation of p-coumaroyl-CoA (derived from the PAL pathway) with three molecules of malonyl-CoA [18]. The product of this reaction is a chalcone (naringenin chalcone), which is converted to isoflavone (as genistein) by other subsequent enzymes, such as Chalcone Reductase (CHR) and Isoflavone Synthase (IFS).

In multivariate analyses, an association was noted between WUE and genistein with clone C5, suggesting that this genotype responds more efficiently under water-limited conditions, both functionally and metabolically. During stress, plants undergo ROS production, and as a mechanism to regulate this stress, flavonoids interfere, providing a certain type of protection to the plant [19]. This behavior also explains the greater association of A and E with clone C4, revealing the maintenance of high photosynthetic performance despite low flavonoid accumulation. This suggests that when these materials have a high metabolic rate, they are not under stress to produce large amounts of flavonoids or have lower sensitivity to stress, allowing them to accumulate flavonoids. Flavonoids participate in responses to drought stress at various levels, including signal transduction, gene expression regulation, ROS elimination (as mentioned above), stomatal movements, and retention of photosynthetic system functionality, ultimately improving plant performance under drought stress conditions [19].

Irrigation management can be a strategic tool for modulating not only physiological performance but also the content of bioactive compounds in eucalyptus plants. Intermediate irrigation intervals favored a balance between physiological productivity and isoflavone synthesis, especially in clones C1 and C3.

## 4. Materials and Methods

The experiment was conducted at the experimental area of the Federal University of Mato Grosso do Sul (UFMS), Chapadão do Sul Campus. The area is located at an average altitude of approximately 803 m above sea level. According to the Köppen climate classification, the local climate is a tropical humid type (Aw), characterized by a rainy season that extends from October to April and a dry season from May to September.

**Figure 15:**
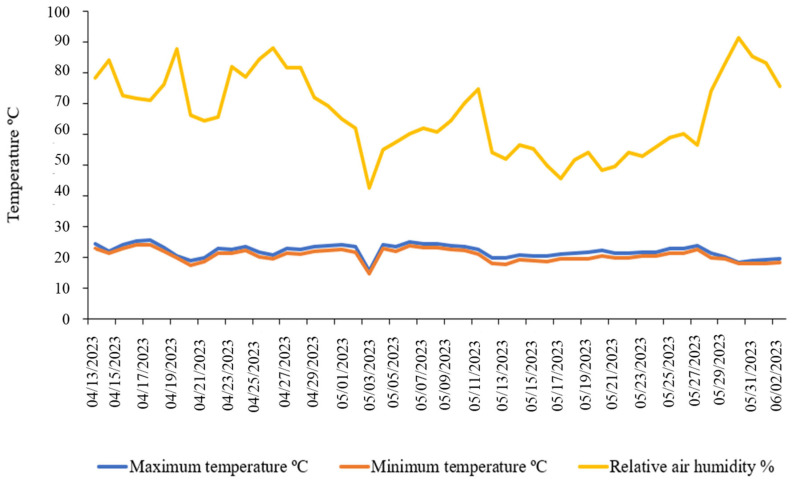
Maximum and minimum temperature values and relative air humidity recorded during the experimental period under greenhouse conditions.

The experiment was carried out in a protected environment, consisting in a 4 × 6 m greenhouse covered with transparent plastic and with the sides closed by a white anti-aphid screen, allowing ventilation and partial control of microclimatic conditions. combined with transparent plastic, which allowed for control of environmental conditions and exclusion of rain interference. Eighty 10 L plastic pots were used in order to focus on only in the initial growth of the plants, in which there is no significant root growth. The pots were prepared by placing an approximately 5 cm layer of gravel at the bottom, followed by a Bidim geotextile fabric to prevent mixing between the drainage layer and the soil. The soil was classified as a medium-textured Red Latosol (Oxisol), according to the referenced classification system [20]. During the experimental period, the average maximum temperature was 22.3 °C, the average minimum temperature was 20.5 °C, and the average relative air humidity was 68.6% (Figure 15).

The experimental design was structured in strips, each containing five seedlings of distinct eucalyptus clones, identified as AEC 0144 (C1), A 217 (C2), ELD01 (C3), ELD08 (C4), and ELD07 (C5), provided by the company Eldorado Brasil (Três Lagoas, Brazil). Irrigation was carried out with a fixed volume of 4 L per application as standardized by companies in the sector, using different irrigation intervals of 1, 2, 4, and 8 days with four replicates for each treatment. Initially, all pots were under the same soil moisture conditions. The irrigation interval lasted 8 days. Nine days after the end of the irrigation interval, physiological evaluations were performed on seedlings of all clones, consistent with procedures used by commercial eucalyptus producers, given that transplant establishment is known to occur within the first few days. Assessments were conducted on the ninth day—one day after irrigation of the daily irrigation treatment—so that all treatments were under comparable conditions at the time of data collection.

Measurements were made using a portable photosynthesis analyzer of the IRGA (Infrared Gas Analyzer) type, model Li-6400XT (LI-COR Inc., Lincoln, NE, USA) (Image 1). The equipment was adjusted with a photosynthetic photon flux density (PPFD) of 1044 μmol m^−2^ s^−1^ and an ambient CO_2_ concentration of 372 ± 10 μmol mol^−1^, as described by Ref. [21]. The leaves evaluated were young and fully developed, with one leaf per plant assessed on two consecutive days, always between 08:00 and 11:00 and between 14:30 and 16:00, under favorable climatic conditions and a clear, cloudless sky and the readings were taken 24 h after each treatment application as performed by [21].

The physiological variables analyzed included the net photosynthetic rate (A, μmol CO_2_ m^−2^ s^−1^), the transpiration rate (E, mmol H_2_O m^−2^ s^−1^), and the instantaneous water use efficiency (WUEi, μmol CO_2_ mol^−1^ H_2_O), calculated as the ratio of A to E. At the end of the physiological analyses, the aerial parts of the seedlings were collected, dried in a forced-air oven at 65 °C for 72 h, and subsequently ground. Subsamples of 0.05 g of dry material were placed in Eppendorf tubes for flavonoid analysis.

**Figure 16:**
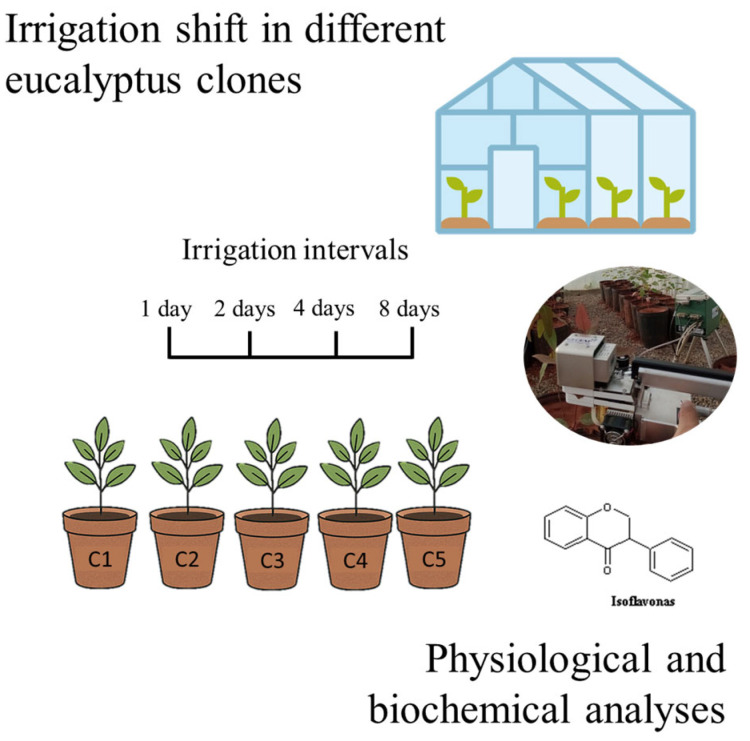
Schematic representation of the experimental workflow.

An aliquot of 1.5 mL of 70% (*v*/*v*) methanol containing 0.1% acetic acid were added in Eppendorf microtubes containing 0.05 g of dry sample. The samples were subjected to ultrasonication (300 W) for 2 h at room temperature, and then centrifuged to obtain the extract. The supernatant was filtered and analyzed by ultra-performance liquid chromatography (UPLC). Chromatographic separation was performed on an HSS C18 column using a binary gradient system with the following solvents: A (water + 0.1% acetic acid) and B (acetonitrile + 0.1% acetic acid). Detection was carried out at 254 nm. Power ultrasound 400 W at room temperature. Scanning was performed 200–400 nm and data processed at 254 nm. For the separation of isoflavones, a binary linear gradient system will be used, with the following mobile phases: Milli-Q water and 0.1% acetic acid as solvent A and acetonitrile and 0.1% acetic acid as solvent B. The initial gradient will be 99% for solvent A and 1.0% for solvent B from 0 to 9 min, 41.2% A and 58.8% B from 9 to 9.1 min, 100% B from 9.1 to 11 min, returning to 99% A and 1% B at 11 min and remaining like this until 15 min, which was the run time for each sample. The mobile phase flow rate will be 0.289 mL min^−1^ and the column temperature during the run will be 30 °C. Reversed-phase column type HSS C18, 1.8 µm (2.1 mm inner diameter (i.d.) £100 mm) with an Acquity HSS C18 pre-column, 1.8 µm (2.1 mm i.d. £5 mm). but details on the methodology: [22]. For compound identification and quantification, commercial standards of isoflavones—daidzein (D1), genistein (G1), and genistin (G2)—were used, based on retention times and UV absorption spectra (Supplementary File S1). Figure 16 provides a schematic representation of the experimental workflow.

Data were subjected to analysis of variance (ANOVA) using Rbio software version 162 (Viçosa, Brazil) [23]. In cases where a significant interaction between factors (clones x irrigation days) was observed, means within each factor were analyzed separately. When no significant interaction was detected, the main effects were analyzed individually. Comparisons among clones were performed using the Scott–Knott test [24] adopting a 5% significance level. For variables related to irrigation management, linear regression analysis was applied at the same significance level. In addition, multivariate analyses, including canonical variable analysis and Pearson’s correlation, were conducted to evaluate the relationships among physiological variables, flavonoid levels, and the different irrigation treatments and clones.

## 5. Conclusions

Eucalyptus clones exhibit distinct physiological and biochemical responses to variation in irrigation intervals. More frequent irrigation favored photosynthetic activity and transpiration, particularly in clones C1, C2, and C3. The joint evaluation of physiological traits and flavonoid profiles revealed a synergistic relationship between these variables under water-stress conditions. In clone C1, genistin accumulation was associated with greater photosynthetic stability, suggesting that this compound helps maintain photosynthetic apparatus functionality via antioxidant activity. In clone C3, increased daidzein content under longer irrigation intervals was linked to enhanced drought tolerance, indicating a role in mitigating oxidative stress and maintaining cellular homeostasis.

These findings indicate that eucalyptus responses to water deficit are genotype dependent and mediated by both physiological adjustments and the biosynthesis of antioxidant compounds. The integration of these mechanisms underscores the importance of flavonoids in protecting photosynthetic processes and provides a biochemical basis for selecting clones with superior resilience and bioactive potential under variable water regimes.

Integrating physiological and biochemical characteristics, as investigated here, can substantially support breeding programs and more sustainable management practices by guiding the selection of genetic materials tailored to specific environments, thereby enhancing adaptation. Expanding the number of tested genotypes, extending experiment duration, and incorporating graded intensities of water stress—together with molecular and agronomic analyses—may yield a more comprehensive understanding of plant behavior across irrigation intervals.

Such approaches will deepen insight into plant responses to water deficit and help identify superior genotypes in terms of both physiological performance and production of bioactive compounds.

## Figures and Tables

**Table 1:** Analysis of variance with mean squares for the variables net photosynthesis (A, μmol CO_2_ m^−2^ s^−1^), transpiration (E, mmol H_2_O m^−2^ s^−1^), instantaneous water use efficiency (WUE, μmol CO_2_ mol^−1^ H_2_O), and the isoflavones daidzein, genistein, and genistin.

FV	DF	A	E	WUE	Daidzein	Genistein	Genistin
Clone	4	112.26 *	32.69 *	15.04 *	92,822 *	28,940 *	9,672,605 *
Error a	12	24.28	3.944	1.38	1228	5434.1	1,221,290
irrigation	3	28.47 *	8.756	2.64 *	15,823	5274.9	2,269,437
Error b	9	5.425	2.302	0.58	2593	2260.5	874,930
Clone X irrigation	12	19.19 *	4.97 *	1.67	14,519 *	9053.0 *	4,035,972 *
Error c	36	6.816	1.967	0.93	2791	4279.6	1,719,021

* Significant 5% by the F test. DF: degrees of freedom.

## Data Availability

The data presented in this study are available on request from the corresponding author. The data are not publicly available due to the fact that they will be used in research project reports.

## Supplementary Materials

The following supporting information can be downloaded at: https://www.mdpi.com/article/10.3390/plants14213405/s1, Supplementary File S1. Chromatography.
