# Supplementary figures and images for: Unveiling the Influence of the Antioxidant System in Eucalyptus Seedlings in the Face of Adequate Water Availability
Source: Plants (Basel). 2025 Nov 6;14(21):3405. doi: 10.3390/plants14213405 (PMC12609279; doi:10.3390/plants14213405)

Supplementary File S1. Chromatography.

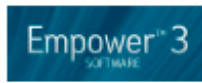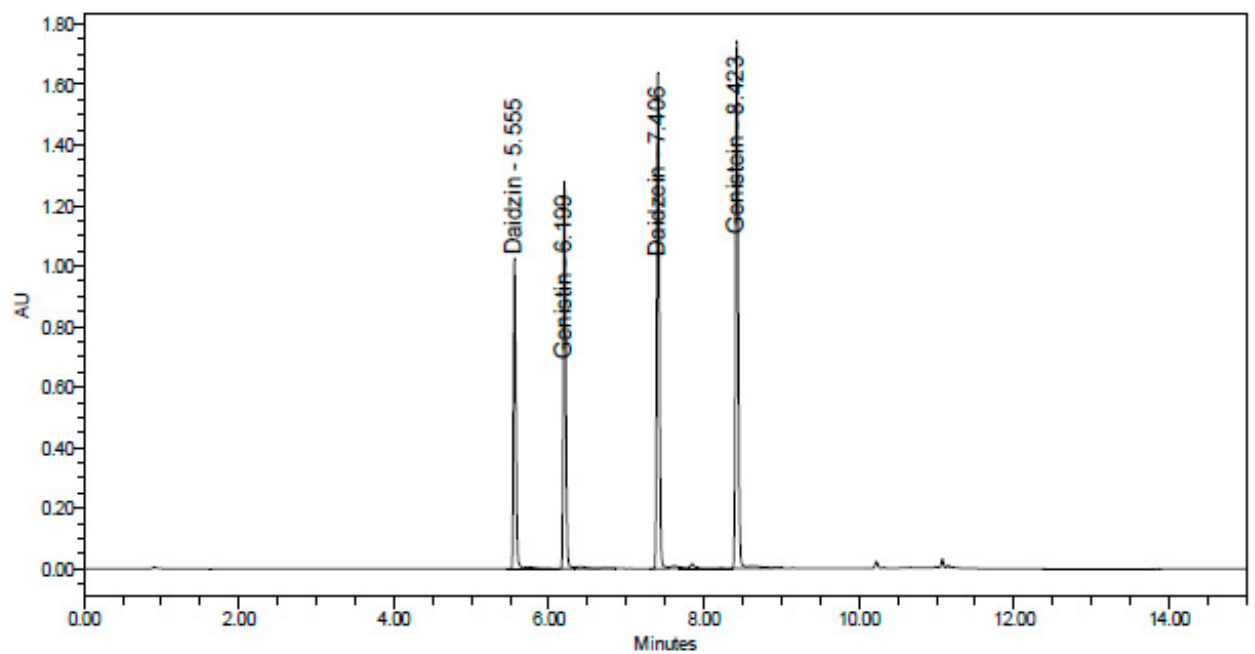

Supplement: Supplementary file 1 [file plants-14-03405-s001.zip › plants-3924097-supplementary.pdf]
